# Impact on allergic immune response after treatment with vitamin A

**DOI:** 10.1186/1743-7075-6-44

**Published:** 2009-10-23

**Authors:** Victor Matheu, Karin Berggård, Yvelise Barrios, Ysamar Barrios, Maria-Rosa Arnau, Jose M Zubeldia, Maria L Baeza, Ove Back, Shohreh Issazadeh-Navikas

**Affiliations:** 1 Department of Clinical Sciences-Division IV; Lund University, Lund, Sweden; 2 Allergy Service, Hospital Universitario NS Candelaria, S/C Tenerife, Spain; 3 Dermatology & Venereology, Department of Clinical Sciences-Division III Lund University; Lund, Sweden; 4 Immunology Section, Hospital Universitario de Canarias, La Laguna Spain; 5 Unidad de Investigación, Hospital Universitario de Canarias, La Laguna, Spain; 6 Estabulario Central, Universidad de La Laguna, Spain; 7 Allergy Service, Hospital General Universitario Gregorio Marañón, Madrid, Spain; 8 Biotech Research & Innovation Centre, Copenhagen University, Denmark

## Abstract

**Background:**

Vitamin A may have some influence on the immune system, but the role in allergy modulation is still unclear.

**Objective:**

To clarify whether high levels of retinoic acid (RA) affects allergic response *in vivo*, we used a murine experimental model of airway allergic disease.

**Methods:**

Ovalbumin (OVA)-immunization/OVA-challenge (OVA/OVA) and house dust mite (HDM)-immunization/HDM-challenge (HDM/HDM) experimental murine models of allergic airway disease, using C57Bl.10/Q groups of mice (n = 10) treated subcutaneously with different concentrations of all-trans RA (0, 50, 500 and 2,500 ug) every 2-days were used to assess the allergic immune response.

**Results:**

Levels of total and specific-IgE in sera were increased in all groups of RA treated OVA/OVA and HDM/HDM mice. Percentage and total amount of recruited eosinophil in airways by bronchoalveolar lavage fluid (BALF) were significantly enhanced in groups treated with 50, 500 and 2,500 ug of RA compared to non-treated mice. However, the group of mice treated with 2,500 ug had less eosinophil recruitment than the other two groups (50 and 500 ug). In parallel, levels of IL-5 and total IgE in BALF were also significantly diminished in the group treated with 2,500 ug compared to the other 2 groups (50 and 500 ug). Finally, total lung resistance was decreased in group treated with 2,500 ug compared to non-treated mice.

**Conclusion:**

Our results suggest that retinoic acid directly enhances allergic response *in vivo*, but in higher doses may produce of immune suppression.

## Introduction

The incidence of atopic diseases can be considered epidemic [1], although sensitization rate is invariant, the prevalence has peaked in some regions [2] and an increase has been recorded in several developing countries [3]. Some epidemiological studies have found that the highest prevalence for asthma symptoms was mainly in Western countries. These findings have not only raised the possibility of genetic factors but also mainly that environmental factors relating to living conditions in these countries are also important [4], such as westernized life style in which air pollution and the consumption of lots of additives/preservatives can be involved. The presence of enriched foodstuffs with several elements, such as fat-soluble vitamins, is one of the differences between developing and developed countries. Although the benefit has no doubt, several studies have described the possible influence on development of allergic diseases.

The influence of fat-soluble vitamins, such as vitamin D or A, on the immune system have been recently studied [5]. Calcitriol, active metabolite of vitamin D, acts in the immune system through its specific intracellular receptor (VDR) and has been recently shown to have influence in experimental murine models of multiple sclerosis [6], diabetes [7], arthritis [8] and asthma [9] with a shift in Th1/Th2 balance.

Similarly, action of vitamin A, so called retinol, is mediated over antigen presenting cells (APC) through specific intracellular retinoid acid receptors (RAR) [10] and retinoid × receptors (RXR)[11], which are present in the immune cells [12]. It has been recently shown, the effect of retinoic acid in T cells, with a decrease of the T-helper 1 (Th1) immune response and an increase of Th2 immune response *in vitro *[10] and *in vivo *[13]. The vitamin A has shown to suppress [14] and prevent [15] the induction *in vivo *of experimental autoimmune encephalomyelitis (EAE), an experimental model of a Th1 disease. And it has been recently suggested that RAR antagonists may be useful as agents to treat rheumatoid arthritis showing the clinical potential of RAR antagonists in arthritis [16]. Retinoic acid also reduces autoimmune renal injury and increases survival in mice [17]. Finally, some epidemiological studies either in US [18] and in Sweden [19] have shown that vitamin A supplementation within first months of life has been associated with increased risk of asthma and postulated with a Th2 skew.

## Materials and methods

### Animals, immunization and treatment

Groups of pathogen-free female C57BL/10 mice, weight 17-21 g, age 6-7 weeks, were used in the experiments. Mice were fed a standard chow diet [20] containing normal range of vitA/g diet (4 IU/g), and kept in a climate-controlled environment with 12-h cycles of light/dark and sound. All animal care and experimentation were conducted at the *Animal Unit of Medical Inflammation Research *(Lund University, Sweden). Additional experiments were performed at *Universidad de La Laguna *and *Hospital General Universitario Gregorio Marañon *(Spain). All experiments were approved by local Institutional Animal Care and Use Committee in accordance with international protocols of caring animals.

Groups of mice were immunized by intraperitoneal injections of 5-μg *Dermatophagoydes pteronyssinus *(HDM, Alk-Abelló, Spain) complexed with 2-mg aluminum potassium sulphate (Alum, Sigma Chemical Co., St Louis, Mo) on days 0 (D0) and 4 (D4). On D14 and D15, mice were challenged with 5-μg HDM delivered intranasally [21] after slight anesthesia. Additional experiments were performed with chicken egg albumin (OVA, Sigma) plus Alum as described previously [22]. On D16, 24 h after the last allergen exposure, the mice were assessed for lung allergic inflammatory response.

Three days before immunization (D-3), different groups of mice received a subcutaneous injection of 50 μL of PBS-Tween 20 buffer containing 0, 50, 500 or 2,500 μg of all-cis-transretinoic acid (ATRA, Sigma). Subsequently, the injections were also given every second day until the end of experiment as follows: D-1, 1, 3, 5, 7, 9, 11, 13 and 15 [20].

### Local immune response

On day 16 mice bronchoalveolar lavage fluid (BALF) was recruited as described before [22,23]. Mice were deeply anesthetized and trachea was cannulated to perform BALF with 1-ml of PBS buffer. Then, when cells were attached to slides by cytospin and stained, cell counts were determined. The number of recruited cells in pulmonary airways was counted after staining (Diff-Quick, Sigma) 24 h after last challenge. Differential of cells were determined after counting at least 400 cells per slide in a blinded fashion. Single supernatants were used to determine IgE levels and cytokine content by enzyme-immunoassays as described before [22] using monoclonal Ab (anti-IL-4, anti-IL-5, anti-IL-13, anti-IFN-γ (BD Pharmingen, San Diego, CA, USA). Standard curves were constructed with purified IL-4, IL-5, IL-13 and IFN-γ.

For RT-PCR analysis, total RNA was extracted with *RNAzol B *(Biotech Laboratories, Friendswood, TX) from homogenized lung cells from right lung, and the oligo (dT)-primed cDNA was prepared with the First-Strand cDNA Synthesis Kit (Amersham Pharmacia Biotech, Buckinghamshire, UK) [24]. To amplify cytokine messages, the samples were incubated as described elsewhere. All PCR reactions were controlled by β-actin expression, and PCR primers as described before [25]; (IL5-6F:5'-AGCACAGTGGTGAAAGAGACCTT; IL5-6R:5'-TCCAATGCATAGCTGGTGATTT) were used as described before [26]. A mathematical model published before was applied [27] and the relative expression ratio was calculated from the real-time PCR efficiencies and the crossing point deviation of an unknown sample versus a control Ct IL-5 represents cycles when IL-5 expression is augmented; Ct Ract represents cycles when R actine expression is augmented (control); Ct IL-5/Ct Ract represents ratio. Control levels were included in the model to standardise each reaction run with respect to RNA integrity, sample loading and inter-PCR variations. High accuracy and reproducibility (<2.5% variation) were reached in LightCycler PCR (Roche, Mannheim, Germany) using the established mathematical model [27].

### Lung responsiveness

In additional experiments, 24 hours after the trans-nasal challenge with OVA, airway responsiveness was assessed. Four individual whole-body plethysmograph chambers, obtained from Buxco (Troy, NY, USA), were used as described elsewhere [28]. In this system, unrestrained, spontaneously breathing mice are placed into the chambers. The pressure differences between these and their respective reference chambers are recorded and then the enhanced pause (Penh) calculated. Penh is a dimensionless value that represents a function of the proportion of maximal expiratory to maximal inspiratory box pressure signals and the timing of expiration. Penh was used to monitor airway responsiveness in this study because it closely correlates to pulmonary resistance measured by conventional ventilated two-chamber plethysmography in mice [28]. Mice were exposed for 2 min to nebulized PBS [29] and subsequently to increasing concentrations of nebulized metacholine (Mch) (Sigma) in PBS using an aerosonic ultrasonic nebulizer (DeVilbiss, PA, USA). After each nebulization, three minutes recordings were taken. Penh measurements were averaged and are expressed for each Mch concentration as the percentage of baseline Penh values following PBS exposure. Three-min recordings of noninvasive measurement of airway responsiveness in allergic mice after each nebulization were assessed [30].

### Systemic immune response

Mice were bled at the time of sacrifice. Total IgE levels in sera and BALF were determined by a sandwich ELISA (BD Pharmingen). OVA-specific IgE levels were measured as described previously [22].

Spleens were dissected at the time of sacrifice and splenocytes were prepared, supplemented and cultured as described before to measured specific cell proliferation [31]. Single cell suspensions from each mouse were prepared with Dulbecco's MEM with glutamax I (GIBCO BRL, Life Technologies), supplemented with 10% heat-inactivated FCS, 10 mmol/l HEPES, 50 mmol/l β-mercaptoethanol, 100 U/ml penicillin G, and 100 μg/ml streptomycin as culture medium. Cells were incubated (5 × 10^6^/ml) in triplicates at 37°C and 5% CO2 in a humidified incubator. Medium, concavalin A as unspecific stimulation or OVA (111 μM) [22] as specific stimulation were separately added to the cultures. 54 hours later, 5 μL of ^3^H-thymidine (100 μCi/ml) were added to each well before harvesting, and its incorporation was measured 18 h later in a beta-scintillation counter [20].

### Statistic analysis

The significance of changes was evaluated using Mann-Whitney *U *test. Significance was assumed at *p *values ≤ 0.05.

## Results

### Cell profile in airways after treatment with all-transretinoic acid has dose-dependent effect

**Figure 1:**
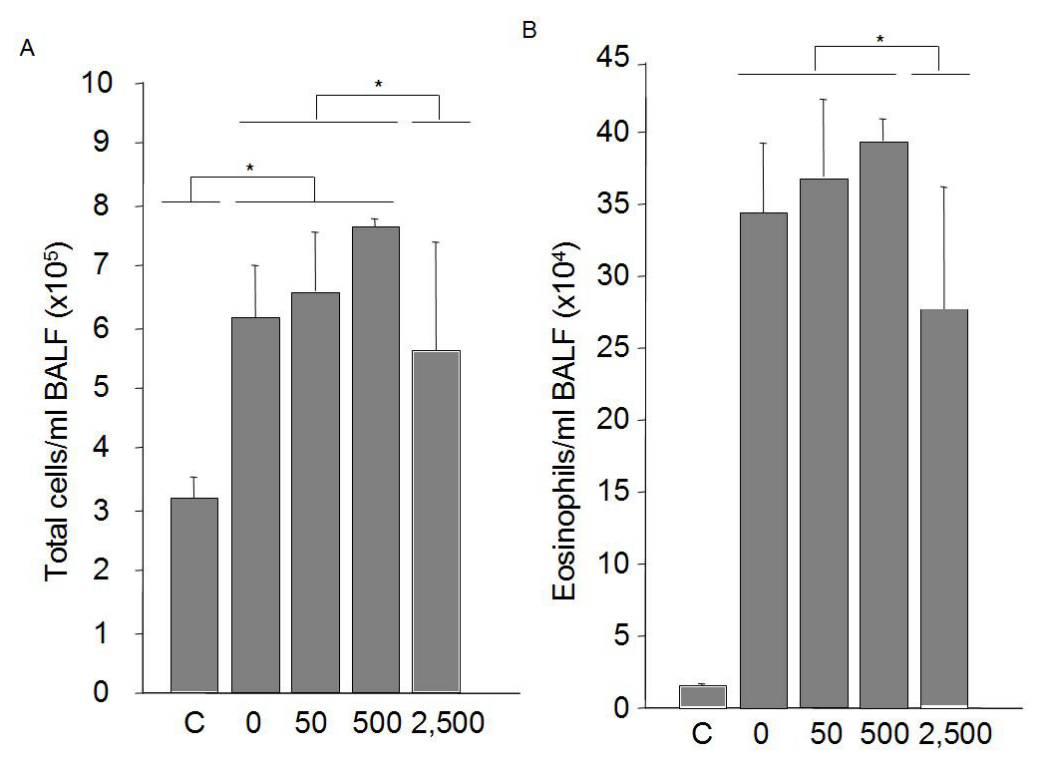
**Cell profile in BALF in OVA-immunized/OVA-challenged mice**. Individual BALF were collected 24 h after the last challenge from each group (*n *= 8/group) and cells were attached to slides, stained with histochemistry staining for eosinophils and, at least 400 cells, counted in a blind manner. Data are given as mean ± SEM, *p < 0.05 vs. non-treated group. † *P *< 0.05 vs 2,500 treated group.

To determine whether administration of different doses of ATRA during all protocol could have any influence in the development of allergic airway phenotype, BALF was collected in every mouse. Total cell count was not significantly affected by doses of 50 and 500 ug ATRA treatments in the murine allergic model. Only when 2,500 ug of RA was administered to mice, the number of recruited cells was affected (table 1). However, the cell profile was different depending on doses of treatment. After treatment with 50 ug ATRA every second day during the protocol, mice had a different profile of recruited cells, with significantly higher percentage of eosinophils (47,8% compared to 39,9% in non-treated) and lymphocytes and less macrophages and neutrophils in airways than HDM/sensitized-HDM-challenge mice treated with control buffer during the protocol (table 1). The percentage of eosinophils was almost 50% in group treated with 500 ug and finally, it was reduced to 11% in group treated with 2,500 ug every second day. Similar differences were obtained when we performed immunization and challenge using OVA (Figure 1).

**Table 1:** Profile of recruited cells from airways by BALF

**Treatment**	**Total cells**	**Eosinophils**	**Monocytes**	**Lymphocytes**	**Neutrophils**
PBS	240 ± 38§	3 ± 1§	201 ± 18	8 ± 5§	10 ± 7

Dpt+Vit A 50	612 ± 36	293 ± 28	198 ± 24	86 ± 15	34 ± 8

Dpt+Vit A 500	624 ± 59	312 ± 29	199 ± 22	82 ± 12	29 ± 7

Dpt+Vit A 2500	428 ± 46	46 ± 35*	284 ± 34	79 ± 11	19 ± 7

Cells from BALF of house dust mite-sensitized and house dust mite challenge mice treated with ATRA or control buffer during the entire protocol. §p < 0.05 compared to groups treated with 50 and 500 of ATRA; * p < 0.05 compared to groups treated with 50 and 500 of ATRA.

### Th2 cytokine induction and total IgE in airways by all transretinoic acid

**Figure 2:**
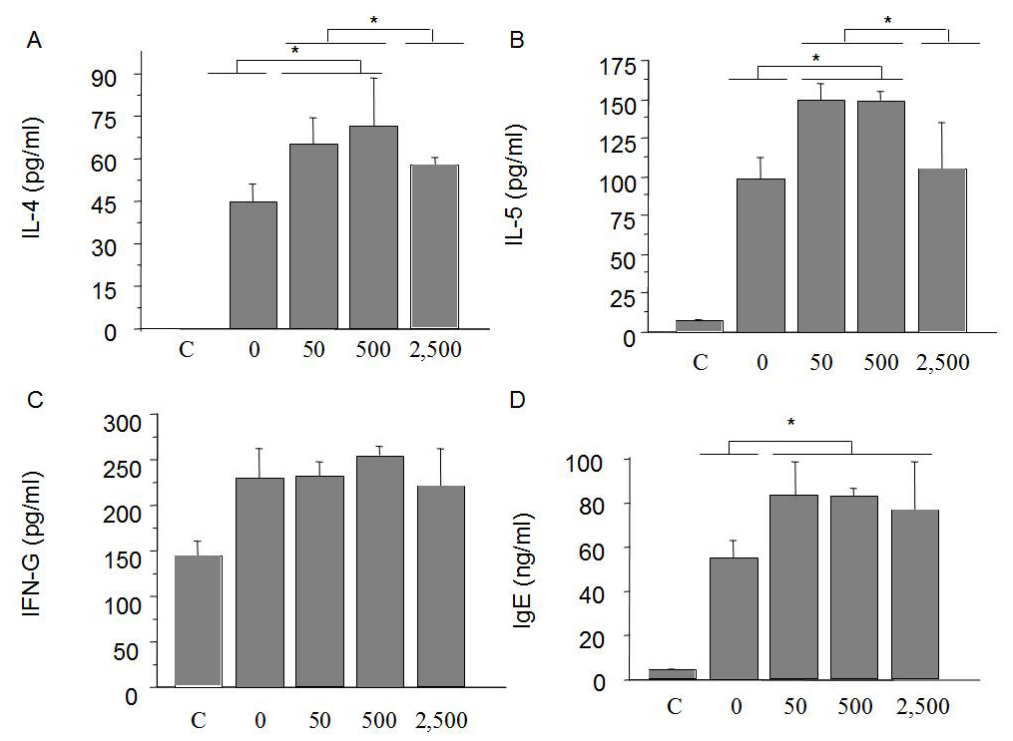
**BALF cytokine levels**. Concentration of protein determined by ELISA in immunized and challenged mice 24 h after last intranasal allergen challenge (*n *= 8/group). (A) IL-4 levels were significantly augmented in all treated groups compared to non-treated group. (B) IL-5 levels were significantly augmented in groups treated with 50 and 500 ug of VA every second day compared to non-treated group. However, levels in group of mice treated with 2,500 ug every second day, were reduced compared to previous treated groups. There was not significant difference between 2,500 group and non-treated group. (C) IFN-γ in airways was not modified in treated mice. (D) Total IgE content in airways was also significantly increased after treatment with RA in all groups compared to non-treated mice. Data are given as mean ± SEM, *P < 0.05 vs. non-treated group.

Then, we studied the results of Th2 cytokines affected by the minimal dose of RA treatment. Supernatants of BALF were thawed and used to determine the airway cytokine and IgE levels contents. We observed that the levels of IL-4 production (Figure 2a) in BALF were enhanced upon 50, 500 or 2,500 ug of ATRA treatment (50 ug; mean: 67 ng/ml; 500 ug: 71 ng/ml; 2,500 ug: 59 ng/ml) compared to control buffer treated allergic mice (0 ug; mean: 44 ng/ml; p < 0,05 n = 8 mice per group).

IL-5 (Figure 2B) was also enhanced in groups treated with 50 and 500 ug ATRA (50 ug; mean: 151 ng/ml; 500 ug: 149 ng/ml) compared to non-treated mice (0 ug; mean: 98 ng/ml). However, the group of mice treated with 2,500 ug (2,500 ug; mean: 106 ng/ml) have lower levels of IL-5 than the other 2 groups (50 and 500 ug; p < 0,05) but were not significantly different from non-treated mice.

IFN-gamma production in airways of mice was similar in all groups (0 ug; mean: 228 ng/ml; 50 ug: 231 ng/ml; 500 ug: 252 ng/ml; 2,500 ug: 221 ng/ml) compared to non-allergic control mice (mean: 148 ng/ml) (Figure 2c).

Similarly to IL-4 results, levels of total IgE in airways of mice were enhanced upon treatment in all treated groups (50 ug; mean: 84 ng/ml; 500 ug: 82 ng/ml; 2,500 ug: 77 ng/ml) compared to non-treated allergic mice (0 ug; mean: 57 ng/ml) (Figure 2d).

### Total and specific IgE are increased after treatment with all-transretinoic acid

**Figure 3:**
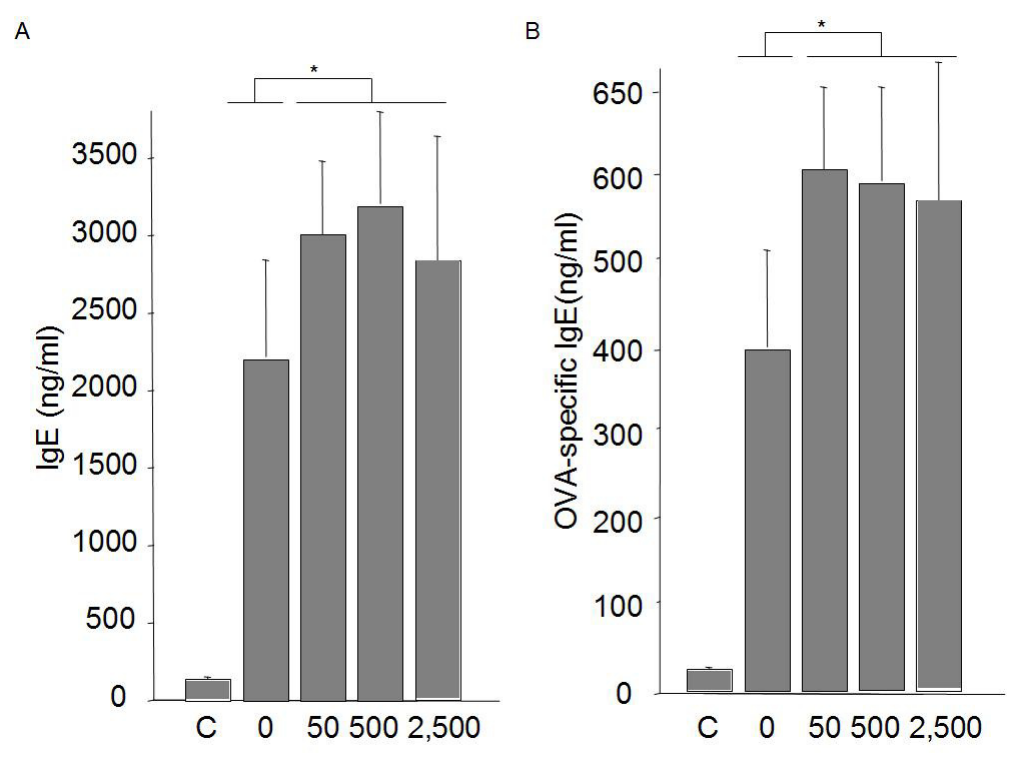
**Levels of total and specific IgE**. Levels were determined by sandwich ELISA in individual sera from immunized and challenged mice (*n *= 8/group) A) Total IgE levels showed increased levels in groups treated with ATRA compared to control mice received PBS alone. Significant differences were observed between treated and non-treated groups.

Next, we investigated whether treatment with ATRA every second day had an influence on the IgE response at the end of protocol. We found that treatment with ATRA resulted in significant enhancement of total IgE in all groups (50 ug; mean: 3,020 ng/ml; 500 ug: 3,189 ng/ml; 2,500 ug: 2,897 ng/ml) compared with non-treated group (0 ug; mean: 2,149 ng/ml; Figure 3a). Specific IgE was also increased by ATRA treatment (50 ug; mean: 603 ng/ml; 500 ug: 591 ng/ml; 2,500 ug: 578 ng/ml) compared with non-treated mice (0 ug; mean: 399 ng/ml; Figure 3b).

### Specific spleen cell proliferation was not affected by *in vivo *treatment with all-trans retinoic acid

**Figure 4:**
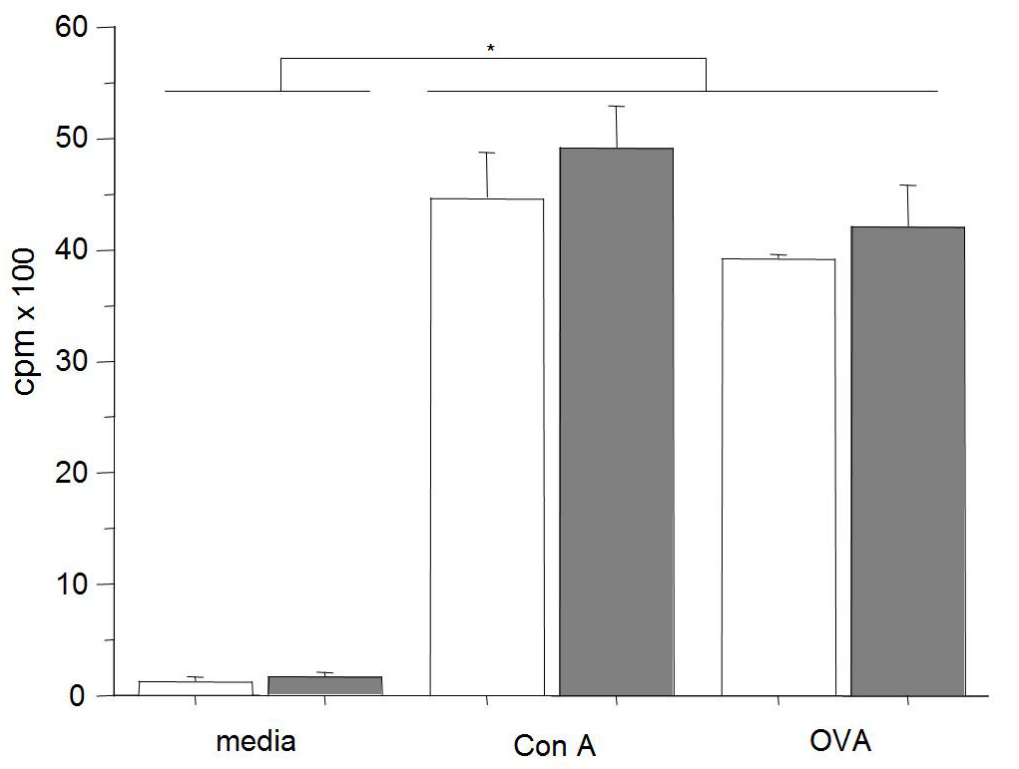
***Ex-vivo *proliferation assays**. Splenocytes from individual treated and non-treated mice (*n *= 5/group) were harvested on day 17 after immunization and challenge and cultured in triplicates in 96-well flat-bottomed plates at 37°C, 5% CO2 and stimulated *in vitro *in absence or presence of OVA (111 μM). ^3^H-thymidine (100 μCi/ml) was added to cultures 2 days later and after 18 hr proliferation was measured by a beta-scintillation counter. No differences were observed between treated and non-treated groups of mice.

We were interested to investigate how the dose of ATRA affects the systemic specific T cell proliferation. After lysis of RBC, single cell suspensions of splenocytes from each mouse treated with ATRA were cultured in triplicates in absence or presence of OVA. Measurement by a beta-scintillation counter showed that antigen-specific *in vitro *cell proliferation was not affected by *in vivo *treatment of mice with 50 ug of ATRA every second day (Figure 4).

### Treatment with highest dose of retinoic acid abolished airway hyperresponsiveness and downregulated expression of IL-5

**Figure 5:**
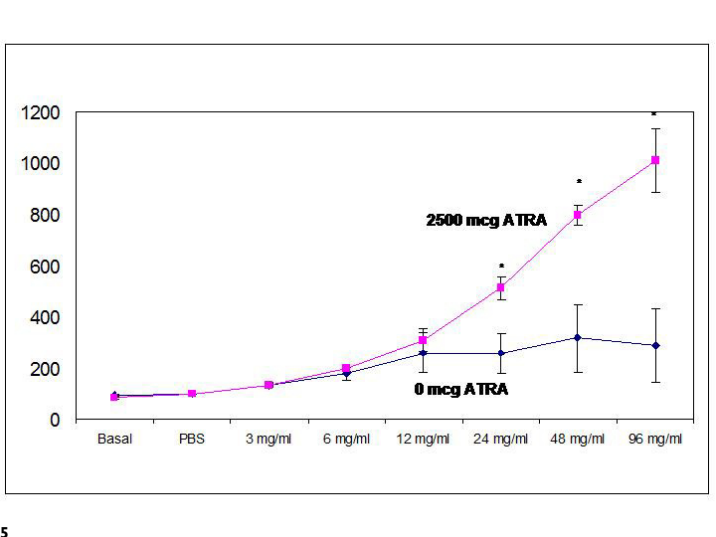
**Determination of airway hyperresponsiveness**. Pulmonary function test were performed in sensitized and transnasally challenged mice. Mice were exposed to increasing doses of inhaled methacholine (Mch) and Penh values were registered. Allergic mice treated with 2,500 mcg of vit A developed a significantly decreased response to Mch when compared to allergic untreated controls. Values are expressed as mean ± SEM (n = 6 mice per group) for each Mch dose point. * p < 0,05.

Since cell profile in airway resulted in amelioration of eosinophils and levels of IL-5 in airways when highest doses of ATRA (2,500 ug every second day) were applied in mice, we then were interested in investigating if studies of airway function *in vivo *was affected. Additional experiments were performed using the same protocol -OVA/OVA- and 24 h after the last intranasal challenge with OVA, ATRA treated mice showed a significant 2 to 3-fold decrease of the airway response to metacholine using whole-body plethysmography, when compared to the untreated mice, as demonstrated by a progressive decrease in P-enh values (Figure 5). Significant enhanced pause (P-enh) was obtained at 24, 48 and 96 mg/mL of Mch inhalation. This result confirmed that treatment with the highest dose of ATRA every second day is enough to suppress not only eosinophil recruitment in airways but also functional effects on the airways.

**Figure 6:**
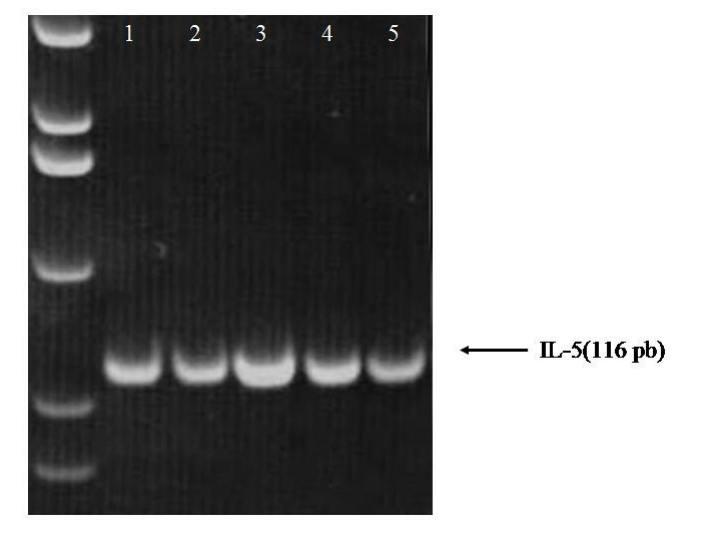
**RT-PCR of IL-5**. For qualitative assessment, the PCR products were analyzed on 5% acrylamide gels stained with ethidium bromide. For quantitative analysis of IL-5, we used the Light Cycler quantitative PCR system. Based on the standard values of the control samples, the relative value for each test sample was determined with the Light Cycler software. In line 1 controls. In lines 2 & 3 treated allergic mice. In lines 4 & 5 non-treated allergic mice.

In additional experiments, when the highest doses of ATRA every second day were applied in mice, RT-PCR analysis of homogenized lung parenchyma cells was also done. After total RNA was extracted, the oligo (dT)-primed cDNA was prepared. For qualitative assessment, the PCR products were analyzed on 5% acrylamide gels stained with ethidium bromide. As the figure 6 shows, bands of IL-5 were more intense in mice treated with 2,500 ug of ATRA every second day compared to non-treated mice. In quantitative analysis of IL-5, using the Light Cycler quantitative PCR system (Roche, Mannheim, Germany), results shows that there is a significant reduction of IL-5 expression in lung parenchyma of non-treated allergic mice compared to mice treated with the highest dose of ATRA every second day.

## Discussion

Fat-soluble vitamins, such as vitamin D or A, are common food additives with some influence on immune system. The active metabolite of vitamin D, calcitriol, has been shown to prevent the induction of experimental models of autoimmune diseases and could influence the development of a sustained Th2 response [9], leading to an increasing prevalence of allergy.

Similarly, action of vitamin A, so called retinol, is mediated over antigen presenting cells (APC) through specific intracellular receptors (RAR, RXR) [10,11], which are present in the immune cells. It has been recently shown that the effect of retinoic acid in T cells [32] might produce a decrease of the Th1 immune response [12] and an increase of Th2 immune response in vitro [10]. Vitamin A in the body is acquired from diet as preformed vitamin A (mainly retinyl ester form) but also less amount of retinol or retinoic acid [33]. Vitamin A is also acquired as dietary pro-vitamin A carotenoids, which are absorbed by the mucosal cells and are converted to retynaldehyde. Upon reduction to retinol, this is indistinguishable from other forms coming from diet. For biological activity, retinol must be oxidized to retinaldehyde and, subsequently to retinoic acid [33]. In the present work, we used all-trans retinoic acid by subcutaneous administration trying to avoid some gastrointestinal interference in absorption of vitamin A, and having of stable levels of retinoic acid in blood. Retinoic acid has an important role in embryo development (hindbrain and associated neural crest) with mid-gestation interruption in retinoic total deficiency [33]. Thus, the goal of our model was not to be deficient in vitamin A in control mice, since RA deficiency is rare in western countries.

Our results show that treatment with some extra-supplementation of ATRA produces an up-regulation of IgE in serum. It is known that the balance between Th1 and Th2 cells is in part controlled by Vitamin A, which inhibits IL-12 synthesis through binding to retinoid receptors, thereby favouring a Th2 response [12]. Other authors have shown that blocking the RXR subunit of retinoid receptors augmented the production of IL-12 by activated monocytes and inhibited the development of Th2 cells in vitro. Furthermore, in a mouse model of allergic asthma, treatment with an RXR antagonist decreased the antigen-specific Th2 response leading to reduced serum IgE levels and strongly decreased lung eosinophilia after antigen challenge. Therefore, retinoid receptor antagonists hold promises for the treatment of Th2-mediated diseases [34,35]. Furthermore, in a prospective birth cohort of 4089 newborn infants followed for 4 years using parental questionnaires, children supplemented with vitamins A and D in water-soluble form during the first year of life had an almost 2-fold increased risk of asthma and sensitization to common food and airborne allergens at age 4 years compared with those receiving vitamins in peanut oil, suggesting that supplementation of vitamins A and D in water-soluble form seemed to increase the risk of allergic disease [19].

Previously, some authors have shown that ATRA inhibits IgE synthesis from anti-CD40 plus IL-4 stimulated human B lymphocytes in in vitro experiments. Furthermore, retinoic acid inhibited CD40 plus IL-4 mediated-IgE production through alterations of sCD23, sCD54 and IL-6 production [36]. And mechanisms of strengthen humoral immunity by promoting CpG-mediated stimulation of CD27(+) B cells via activation of p38MAPK have been suggested [37]. Maybe, as Gottesman et al have postulated, *in vitro *data are individually important but the metabolic pathways are important also *in vivo*, since the situation *in vitro *does not reproduce the complexity of normal physiology *in vivo *and, in particular, the known plasticity in retinoid transport pathways [33].

We have also shown that retinoic acid up-regulates airway eosinophils. Some authors have compared the effect of a treatment with intraperitoneal injections with liposomally encapsulated retinoic acid in a mouse model of ovalbumin. They show exacerbation of allergic immune and inflammatory responses, most likely by promoting Th2 development [38]. Very recently, some authors have shown that Vitamin A deficiency (VAD) can produce a Th1 bias, whereas high-level dietary vitamin A can promote a Th2 bias using an OVA exposure mouse model. VAD reduced serum IgE and IgG1 responses, pulmonary eosinophilia, and the levels of IL-4 and IL-5 in BALF specimen [39]. Same authors also showed that it was a different result comparing sub-total and total deficiency of VA [39]. Possibly, in times of inadequate vitamin A intake, retinol binding protein RBP ensures the retinol is available for maintaining normal cellular functions [33]. However, in cultures of normal human bone marrow, ATRA selectively suppressed eosinophil differentiation. Similarly, ATRA inhibited eosinophil/basophil differentiation of cord blood CD34+ cells, while neutrophil differentiation proceeded without impediment [40].

Surprisingly, when the highest dose of RA was applied, percentage of eosinophil was reduced. In concordance with results of airway eosinophilia, levels of IL-5 expression in parenchyma cells and lung function were also abolished by treatment with highest doses of retinoic acid. It might be explained either by an immunosuppressive effect as postulated by others since retinoic acid seems to down-regulate expression of the cutaneous lymphocyte-associated antigen (CLA), a surface glycoprotein expressed by skin-homing T cells [41]. In a very recent study, Schuster et al. showed that IL-5 was significantly decreased after high dose of vitamin A in mice, which were not fully deficient in that vitamin [39]. All these results might demonstrate that in studies with fat-soluble vitamins results of in vitro tests should be taken carefully.

However, those levels are very difficult to get in human beings since blood levels of retinol bound to RBP are maintained between narrow limits throughout adult life, and change only in response to extremes of vitamin A shortfall and in disease states [33]. However, these results may have some positive application as some agonist of retinoid or antagonist of retinoid receptors might have the ability of prevent eosinophil influx in vivo [35].

In summary, although a supplementation for infants and young children is recommended by WHO for countries with a high prevalence of vitamin A deficiency, that supplementation with vitamin A or other retinoids have the ability of exacerbating the Th2 response, mainly at the time of neonatal immunization [42]. Although in our experiments levels of IFN-gamma were unaffected by treatment with retinoic acid [34], it has been demonstrated that retinoic acid have some influence in Th1 to Th2 balance. As respiratory allergic diseases are characterized by a late-phase immune response in which, Th2 cells recognizing common airborne antigens produce an inflammatory response with some Th2 cytokines IL-4, IL-5, and IL-13, this extra-supplementation in westernised countries might influence the allergy epidemic. The properties of retinoic acid in higher doses could be of therapeutic use, but more studies are still necessary.

## Abbreviations

APC: Antigen presenting cells; DAB: 3.3 diamino benzidine tetrahydrochlorhide; BALF: bronchoalveolar lavage fluid; CREPA: (cyanide-resistant eosinophil peroxidase activity).

## Competing interests

The authors declare that they have no competing interests.

## Authors' contributions

VM conceived and participated in the design of the study, interpretation of data and drafted the manuscript. KB participated in the design of the study, carried out the *in vivo and in vitro *study and interpretation of data. YB participated carried out the *in vitro *study and helped to draft the manuscript. YB conceived in the design, carried out the *in vitro *study, interpretation of data and helped to draft the manuscript. MRA participated and carried out some *in vivo *studies MLB participated in the design, carried out the *in vivo *study, interpretation of data and helped to draft the manuscript. JMZ participated in the design, carried out the *in vivo *study, interpretation of data and helped to draft the manuscript. OB conceived and participated in the design, interpretation of data and helped to original draft the manuscript. SI-N conceived and participated in the design of the study, interpretation of data and helped to draft the manuscript.

## References

[B1] Holgate ST (1999). The epidemic of allergy and asthma. Nature.

[B2] Asher MI, Montefort S, Bjorksten B, Lai CK, Strachan DP, Weiland SK, Williams H (2006). Worldwide time trends in the prevalence of symptoms of asthma, allergic rhinoconjunctivitis, and eczema in childhood: ISAAC Phases One and Three repeat multicountry cross-sectional surveys. Lancet.

[B3] Bjorksten B, Clayton T, Ellwood P, Stewart A, Strachan D (2008). Worldwide time trends for symptoms of rhinitis and conjunctivitis: Phase III of the International Study of Asthma and Allergies in Childhood. Pediatr Allergy Immunol.

[B4] Gold DR, Wright R (2005). Population disparities in asthma. Annu Rev Public Health.

[B5] Camagna A, Testa U, Masciulli R, Barberi T, Samoggia P, Tritarelli E, Pustorino E, Cipollone L, Ciancio L, del Duca P, Dionisi S, del Vecchio LR, Misasi G, de Martinis C, Peschle C (1998). The synergistic effect of simultaneous addition of retinoic acid and vitamin D3 on the in-vitro differentiation of human promyelocytic leukemia cell lines could be efficiently transposed in vivo. Med Hypotheses.

[B6] Lemire JM, Archer DC (1991). 1,25-dihydroxyvitamin D3 prevents the in vivo induction of murine experimental autoimmune encephalomyelitis. J Clin Invest.

[B7] Casteels KM, Mathieu C, Waer M, Valckx D, Overbergh L, Laureys JM, Bouillon R (1998). Prevention of type I diabetes in nonobese diabetic mice by late intervention with nonhypercalcemic analogs of 1,25-dihydroxyvitamin D3 in combination with a short induction course of cyclosporin A. Endocrinology.

[B8] Cantorna MT, Hayes CE, DeLuca HF (1998). 1,25-Dihydroxycholecalciferol inhibits the progression of arthritis in murine models of human arthritis. J Nutr.

[B9] Matheu V, Mondoc E, Back O, Issazadeh-Navikas S (2001). Vitamin D enhances allergic response in mice. Scand J Immunol.

[B10] Iwata M, Eshima Y, Kagechika H (2003). Retinoic acids exert direct effects on T cells to suppress Th1 development and enhance Th2 development via retinoic acid receptors. Int Immunol.

[B11] Stephensen CB, Rasooly R, Jiang X, Ceddia MA, Weaver CT, Chandraratna RA, Bucy RP (2002). Vitamin A enhances in vitro Th2 development via retinoid × receptor pathway. J Immunol.

[B12] Cantorna MT, Nashold FE, Hayes CE (1995). Vitamin A deficiency results in a priming environment conducive for Th1 cell development. Eur J Immunol.

[B13] Matheu V, Barrios Y, Berggård K, Baeza M, Zubeldia J, Back O, Issazadeh-Navikas S (2007). Influence of retinoic acid in allergic inflammation. Ann Allergy Asthma Immunol.

[B14] Massacesi L, Abbamondi AL, Giorgi C, Sarlo F, Lolli F, Amaducci L (1987). Suppression of experimental allergic encephalomyelitis by retinoic acid. J Neurol Sci.

[B15] Racke MK, Burnett D, Pak SH, Albert PS, Cannella B, Raine CS, McFarlin DE, Scott DE (1995). Retinoid treatment of experimental allergic encephalomyelitis. IL-4 production correlates with improved disease course. J Immunol.

[B16] Beehler BC, Hei YJ, Chen S, Lupisella JA, Ostrowski J, Starrett JE, Tortolani D, Tramposch KM, Reczek PR (2003). Inhibition of disease progression by a novel retinoid antagonist in animal models of arthritis. J Rheumatol.

[B17] Kinoshita K, Yoo BS, Nozaki Y, Sugiyama M, Ikoma S, Ohno M, Funauchi M, Kanamaru A (2003). Retinoic acid reduces autoimmune renal injury and increases survival in NZB/W F1 mice. J Immunol.

[B18] Milner JD, Stein DM, McCarter R, Moon RY (2004). Early infant multivitamin supplementation is associated with increased risk for food allergy and asthma. Pediatrics.

[B19] Kull I, Bergstrom A, Melen E, Lilja G, van Hage M, Pershagen G, Wickman M (2006). Early-life supplementation of vitamins A and D, in water-soluble form or in peanut oil, and allergic diseases during childhood. J Allergy Clin Immunol.

[B20] Matheu V, Back O, Mondoc E, Issazadeh-Navikas S (2003). Dual effects of vitamin D-induced alteration of TH1/TH2 cytokine expression: enhancing IgE production and decreasing airway eosinophilia in murine allergic airway disease. J Allergy Clin Immunol.

[B21] Soto-Montenegro ML, Conejero L, Vaquero JJ, Baeza ML, Zubeldia JM, Desco M (2009). Assessment of airway distribution of transnasal solutions in mice by PET/CT imaging. Mol Imaging Biol.

[B22] Matheu V, Treschow A, Navikas V, Issazadeh-Navikas S (2003). Upregulation of B7 molecules (CD80 and CD86) and exacerbated eosinophilic pulmonary inflammatory response in mice lacking the IFN-beta gene. J Allergy Clin Immunol.

[B23] Zuberi RI, Apgar JR, Chen SS, Liu FT (2000). Role for IgE in airway secretions: IgE immune complexes are more potent inducers than antigen alone of airway inflammation in a murine model. J Immunol.

[B24] Chomczynski P, Sacchi N (1987). Single-step method of RNA isolation by acid guanidinium thiocyanate-phenol-chloroform extraction. Anal Biochem.

[B25] Overbergh L, Valckx D, Waer M, Mathieu C (1999). Quantification of murine cytokine mRNAs using real time quantitative reverse transcriptase PCR. Cytokine.

[B26] Overbergh L (1999). Quantification of murine cytokine mRNAs using real time quantitative reverse transcriptase PCR. Cytokine.

[B27] Pfaffl MW (2001). A new mathematical model for relative quantification in real-time RT-PCR. Nucleic Acids Res.

[B28] Hamelmann E, Schwarze J, Takeda K, Oshiba A, Larsen GL, Irvin CG, Gelfand EW (1997). Noninvasive measurement of airway responsiveness in allergic mice using barometric plethysmography. Am J Respir Crit Care Med.

[B29] Alenmyr L, Matheu V, Uller L, Greiff L, Malm-Erjefalt M, Ljunggren HG, Persson CG, Korsgren M (2005). Blockade of CTLA-4 promotes airway inflammation in naive mice exposed to aerosolized allergen but fails to prevent inhalation tolerance. Scand J Immunol.

[B30] Conejero L, Higaki Y, Baeza ML, Fernandez M, Varela-Nieto I, Zubeldia JM (2007). Pollen-induced airway inflammation, hyper-responsiveness and apoptosis in a murine model of allergy. Clin Exp Allergy.

[B31] Matheu V, Treschow A, Teige I, Navikas V, Issazadeh-Navikas S (2005). Local therapy with CpG motifs in a murine model of allergic airway inflammation in IFN-beta knock-out mice. Respir Res.

[B32] Iwata M, Hirakiyama A, Eshima Y, Kagechika H, Kato C, Song SY (2004). Retinoic acid imprints gut-homing specificity on T cells. Immunity.

[B33] Gottesman ME, Quadro L, Blaner WS (2001). Studies of vitamin A metabolism in mouse model systems. Bioessays.

[B34] Grenningloh R, di Lucia P, Gho A, Bollag W, Sinigaglia F, Panina-Bordignon P (2001). Retinoid-receptor antagonists inhibit T helper 2 responses in vitro and in vivo and prevent eosinophil influx into the lung of mice with acute allergic airway inflammation. Scand J Immunol.

[B35] Grenningloh R, Gho A, di Lucia P, Klaus M, Bollag W, Ho IC, Sinigaglia F, Panina-Bordignon P (2006). Cutting Edge: Inhibition of the retinoid × receptor (RXR) blocks T helper 2 differentiation and prevents allergic lung inflammation. J Immunol.

[B36] Scheffel F, Heine G, Henz BM, Worm M (2005). Retinoic acid inhibits CD40 plus IL-4 mediated IgE production through alterations of sCD23, sCD54 and IL-6 production. Inflamm Res.

[B37] Ertesvag A, Aasheim HC, Naderi S, Blomhoff HK (2007). Vitamin A potentiates CpG-mediated memory B-cell proliferation and differentiation: involvement of early activation of p38MAPK. Blood.

[B38] Maret M, Ruffie C, Periquet B, Campo AM, Menevret M, Phelep A, Dziewiszek K, Druilhe A, Pretolani M (2007). Liposomal retinoic acids modulate asthma manifestations in mice. J Nutr.

[B39] Schuster GU, Kenyon NJ, Stephensen CB (2008). Vitamin A deficiency decreases and high dietary vitamin A increases disease severity in the mouse model of asthma. J Immunol.

[B40] Denburg JA, Sehmi R, Upham J (2001). Regulation of IL-5 receptor on eosinophil progenitors in allergic inflammation: role of retinoic acid. Int Arch Allergy Immunol.

[B41] Yamanaka K, Dimitroff CJ, Fuhlbrigge RC, Kakeda M, Kurokawa I, Mizutani H, Kupper TS (2008). Vitamins A and D are potent inhibitors of cutaneous lymphocyte-associated antigen expression. J Allergy Clin Immunol.

[B42] Sankaranarayanan S, Ma Y, Bryson MC, Li NQ, Ross AC (2007). Neonatal-age treatment with vitamin A delays postweaning vitamin A deficiency and increases the antibody response to T-cell dependent antigens in young adult rats fed a vitamin A-deficient diet. J Nutr.

